# First report of multidrug-resistant carbapenemase-producing *Aeromonas caviae* co-harboring *mcr-3.43* and *mcr-7.2*

**DOI:** 10.1128/spectrum.03685-23

**Published:** 2024-03-21

**Authors:** Tingting Xu, Jingjie Song, Jialong Liu, Lili Huang, Zhao Li, Kai Zhou

**Affiliations:** 1 Shenzhen Institute of Respiratory Diseases, Shenzhen People’s Hospital (The Second Clinical Medical College, Jinan University; The First Affiliated Hospital, Southern University of Science and Technology), Shenzhen, Guangdong, China; 2 Department of Clinical Laboratory, Fifth Affiliated Hospital, Southern Medical University, Guangzhou, Guangdong, China; 3 School of Basic Medicine Sciences, Guangxi Medical University, Nanning, Guangxi, China; American Type Culture Collection, Manassas, Virginia, USA

**Keywords:** *Aeromonas caviae*, *mcr-3.43*, *mcr-7.2*, *bla*_KPC-2_, *bla*_NDM-1_

## Abstract

Hospital sewage serves as a crucial reservoir for antibiotic resistance genes. As colistin and carbapenems are the last-resort antibiotics, the emergence of their resistance genes has become a significant concern in clinical settings. In this study, we found that two novel *mcr* alleles (*mcr-3*.43 and *mcr-7*.2) with two carbapenemase genes (*bla*_NDM-1_ and *bla*_KPC-2_) were encoded in a single *Aeromonas caviae* strain isolated from hospital sewage. Our phylogenetic analysis revealed that the *mcr-3.43* gene clustered with *mcr-3.17* (with 95.55% amino acid identity), while the *mcr-7.2* gene clustered with *mcr-7.1* (with 68.68% amino acid identity). BLAST search against GenBank showed that *mcr-7.2* was exclusively detected in *Aeromonas* spp. Mobile genetic elements were not found in the genetic context of *mcr-7.2*, suggesting that the dissemination of *mcr-7.2* in *Aeromonas* spp. may be dependent on vertical transfer or recombination. The *bla*_NDM-1_ was adjacent to a recombinase gene and flanked by two IS*91* elements, indicating a potential mobilization mechanism mediated by recombination and/or ISs. The *bla*_KPC-2_ gene was located on an IncU plasmid and adjacent to an IS*Kpn6*. In summary, our study provides evidence for *Aeromonas* spp. as one of the potential reservoirs of colistin and carbapenem resistance genes.

**IMPORTANCE:**

The study discovered two novel *mcr* genes (*mcr-3.43* and *mcr-7.2*) and two carbapenemase genes (*bla*_NDM-1_ and *bla*_KPC-2_) in a single *Aeromonas caviae* strain retrieved from hospital sewage. Using phylogenetic analysis and comparative data evaluation, the study revealed the genetic relatedness and dissemination potential of the detected resistance genes. With the exclusive discovery that *mcr-7.2* is only present in *Aeromonas* spp. and the lack of mobile genetic elements in its genetic context, there is a strong indication of limited dissemination. The identification of these four resistance genes in a single strain of *Aeromonas* provided valuable insights into their potential presence in this genus. This study revealed that hospital sewage functions as a significant reservoir for antibiotic resistance genes, including colistin and carbapenem resistance genes.

## INTRODUCTION

*Aeromonas* are emerging pathogens capable of colonizing and infecting multiple hosts (1). They are widely known in aquaculture as potentially infectious organisms (2) causing diseases such as septicemia and furunculosis (3). In addition, *Aeromonas* have been isolated from foods, such as vegetables, dairy products, beef, and pork (4, 5). Some species of *Aeromonas* infect humans, causing gastrointestinal and hepatobiliary system infections, bone infections, and septicemia (6–10). An issue of concern is that there has been an increase in the emergence of multidrug-resistant *Aeromonas* spp., particularly those resistant to last-line antibiotics (11).

Colistin is used in medicine as the last-line treatment for human infections caused by Gram-negative *bacilli* (12). Resistance to colistin was thought to be inherent in some bacterial genera until 2016, when the presence of a mobile colistin resistance gene called *mcr-1* was identified in an *Escherichia coli* strain (13). Following this report, an increasing number of isolates of different origins with *mcr* genes and a colistin resistance phenotype have been reported; in addition, resistance to this molecule has been found to be horizontally transferable (14). Several research groups have demonstrated the presence of *mcr* genes in *Aeromonas* (15–17). A total of 6,497 strains collected from 13 provinces in China between 2016 and 2017 were used for *mcr-3* detection, and eight *Aeromonas* isolates classified into different species were confirmed to be *mcr-3* positive (15). The *mcr-5* gene was detected in an *Aeromonas hydrophila* strain isolated from a fecal sample of a backyard pig (16). In addition, *mcr-3/7*-positive *Aeromonas* species have also been reported from a pork sample collected by the USDA Food Safety and Inspection Service (17).

Another major concern is the increasing emergence and rapid dissemination of mobile genetic elements carrying carbapenemase genes, exacerbated by the limited availability of effective therapeutic options. Carbapenemases are commonly classified according to the Ambler classification scheme (18). Class D carbapenem-hydrolyzing β-lactamases, such as OXA-23, OXA-24/40, and OXA-58-like enzymes, are commonly found in *Acinetobacter baumannii*. Recent studies have shed light on the presence of carbapenemases, including KPC-2, KPC-24, NDM-1, and IMP-4, in the genus *Aeromonas*, underscoring their prevalence within this microbial population (19–23). In 2022, two KPC-2-producing *A. hydrophila* strains were characterized from patients in different intensive care units of a Chinese hospital (19). Additionally, KPC-24, which differs from KPC-2 by a single amino acid change at codon 6 (R6P), was first identified in *Klebsiella pneumoniae* in Chile. Subsequently, the presence of the *bla*_KPC-24_ gene was confirmed in an *Aeromonas veronii* strain isolated from hospital sewage in China (20). The first reported *bla*_NDM_-carrying plasmid from *Aeromonas* spp. was isolated from a patient with community-acquired pneumonia (22). Moreover, a multidrug-resistant *Aeromonas caviae* isolate carrying a novel *bla*_KPC-2_-carrying plasmid and an IMP-4-encoding phage-like plasmid was reported in 2022 (23). These findings demonstrate the presence of major antibiotic resistance genes, including *bla*_KPC-2_, *bla*_KPC-24_, and *bla*_NDM_, in *Aeromonas* strains isolated from both patients and hospital sewage, highlighting the potential dissemination of multidrug-resistant bacteria in healthcare settings.

The co-occurrence of MCR- and carbapenemase-producing bacteria has been increasingly reported. In 2020, a study documented the presence of both *mcr-1* and *bla*_NDM-9_ genes in an *Escherichia coli* strain (ST1011) isolated from human intestinal colonization (24). More recently, in 2023, Wu et al. identified 21 *Aeromonas* strains carrying *bla*_KPC-2_ and *mcr* genes (21). These findings underscore the need for caution when using antibiotics that target these resistance mechanisms. Furthermore, it is imperative to strengthen surveillance and control measures to prevent the further dissemination of multidrug-resistant bacteria.

In this study, we characterized an *Aeromonas* strain isolated from hospital sewage that harbors two novel *mcr* genes (*mcr-3.43* and *mcr-7*.2) with two carbapenemase genes (*bla*_KPC-2_ and *bla*_NDM-1_). These compelling results warrant immediate attention to develop effective strategies to combat the widespread dissemination of *mcr* and carbapenemase-encoding genes.

## RESULTS

### Identification and WGS of a colistin- and carbapenem-resistant MDR *Aeromonas caviae* isolate

A total of 400 strains were recovered from LB plates containing 4 mg/L of colistin for the first time, of which 74 strains were further confirmed as colistin resistant by the broth dilution method (Table S1). PCR analysis revealed that 22 isolates harbored *mcr* genes, of which five harbored *mcr-1*, seven harbored *mcr-3*, two harbored *mcr-5*, and eight harbored *mcr-10*. Among the isolates, one *A. caviae*-designated G77 showed *mcr-3* and non-specific bands of *mcr-9* and *mcr-10. A. caviae* G77 was MDR, resistant to ceftazidime (MIC >64 mg/L), cefuroxime (MIC >64 mg/L), cefepime (MIC >64 mg/L), imipenem (MIC = 16 mg/L), meropenem (MIC = 8 mg/L), ciproﬂoxacin (MIC = 32 mg/L), ampicillin (MIC >128 mg/L), cefoperazone (MIC >128 mg/L), chloramphenicol (MIC = 32 mg/L), polymyxin B (MIC = 4 mg/L), colistin (MIC = 4 mg/L), and trimethoprim/sulfamethoxazole (MIC >32/608 mg/L), while susceptible to amikacin (MIC = 8 mg/L), gentamicin (MIC = 4 mg/L), and tigecycline (MIC = 0.25 mg/L).

To detect the presence of novel *mcr* genes in G77, we performed WGS. The sequencing short and long reads were hybridly assembled to obtain no-gap contigs, resulting in a chromosome of 4,8313,750 bp and five contigs with sizes ranging in size from 5,079 bp to 110,200 bp. The contigs were confirmed to be complete by PCR and Sanger sequencing. The isolate was further confirmed as an *A. caviae* strain by KmerFinder (https://cge.food.dtu.dk/services/KmerFinder/). *In silico* mining revealed that *A. caviae* G77 carried 31 ARGs (Table 1), including homologs of two colistin resistance genes *mcr-3.17* (95.55% amino acid identity) and *mcr-7.1* (68.89% amino acid identity), and the carbapenemase genes *bla*_NDM-1_ and *bla*_KPC-2_. The *mcr* genes and *bla*_NDM-1_ were located on the chromosome, while *bla*_KPC-2_ was detected on a 12,135-bp contig. Replicon typing by Mob_Suite detected an IncU replicon on the contig, suggesting that the contig may belong to a plasmid. Two novel KPC-2-producing multidrug-resistant IncU plasmids (pEC2341-KPC, pEC2547-KPC) from *E. coli* in China were reported in 2021 (25). The blast analysis showed that the coverage of pEC2341-KPC, pEC2547-KPC are both 20.82%, compared with the KPC-2-producing IncU plasmid in this study with a threshold at 80% identity. We searched the National Center for Biotechnology Information (NCBI) genome database and found the co-existence of *bla*_KPC-2_ and *mcr-3* in three *Aeromonas* genomes (assembly accession numbers GCA_003925855.2, GCA_009831085.1, and GCA_014169675.1), and of *bla*_NDM-1_ and *mcr-7* in one genome (assembly accession number GCA_017280155.1). However, genomes containing all four genes were not available. We tested 22 *mcr*-carrying isolates for the *bla*_KPC-2_ and *bla*_NDM-1_ genes by PCR and Sanger sequencing; the results showed that four other isolates besides G77 also carry *bla*_KPC-2_ and *bla*_NDM-1_. This is the first time that these four genes have been identified in a single *Aeromonas* strain.

**TABLE 1:** Plasmid replicons and antimicrobial resistance genes detected in G77^*a*^

Contig number	Start	End	Inc type/Gene	Coverage (%)	Identity (%)	Accession number
Plasmid replicons						
1	2,129,158	2,129,607	IncQ2	100	83.78	FJ696404
3	73	633	IncU	100	100	KF623109
5	1888	2667	IncQ1	97.74	82.2	M28829.1
Antimicrobial resistance genes						
1	62,212	62,766	*aac(6')-Ib*	100	99.82	NG_052358.1
1	62,897	63,727	*bla*_OXA-1_	100	100	NG_049392.1
1	63,865	64,497	*catB3*	100	100	NG_047604.1
1	64,582	65,034	*arr-3*	100	100	NG_048581.1
1	69,247	70,107	*bla*_TEM-1_	100	99.88	NG_050145.1
1	70,889	71,764	*bla*_CTX-M-3_	100	100	NG_048979.1
1	73,975	74,814	*sul1*	100	100	NG_048082.1
1	77,749	78,564	*aph(3')-Ia*	100	100	NG_047430.1
1	84,075	84,959	*mph(E*)	100	100	NG_064660.1
1	85,015	86,490	*msr(E*)	100	100	NG_048007.1
1	409,172	410,389	*tet(E*)	100	99.92	NG_048186.1
1	1,220,392	1,220,865	*dfrA1*	100	100	NG_047676.1
1	1250578	1,251,729	*bla*_MOX-12_	100	99.05	NG_049314.1
1	1530396	1,532,011	*mcr-7.1*	98.4	72.74	NG_056413.1
1	2140839	2,141,675	*aph (6)-Id*	100	100	NG_047464.1
1	2,141,675	2,142,502	*aph(3'')-Ib*	100	99.88	NG_056002.2
1	2,147,946	2,149,160	*floR*	100	100	NG_047869.1
1	2,151,663	2,152,478	*sul2*	100	100	NG_051852.1
1	2,428,358	2,429,163	*aadA16*	95.27	99.75	NG_047339.1
1	2,429,195	2,429,749	*aac(6')-Ib*	100	100	NG_052057.1
1	2,430,508	2,431,140	*catB3*	100	100	NG_047604.1
1	2,431,638	2,432,477	*sul1*	100	100	NG_048082.1
1	2,436,145	2,436,510	*ble-*MBL	100	100	NG_047559.1
1	2,436,514	2,437,326	*bla*_NDM-1_	100	100	NG_049326.1
1	2,441,177	2,442,103	*bla*_PER-3_	100	100	NG_049962.1
1	2,447,049	2,447,888	*sul1*	100	100	NG_048082.1
1	2,454,510	2,455,431	*mph(A*)	100	99.67	NG_047986.1
1	2,764,697	2,766,313	*mcr-3.17*	99.63	95.61	NG_060518.1
1	4,801,054	4,801,848	*bla*_OXA-504_	100	98.24	NG_049782.1
3	3841	4722	*bla*_KPC-2_	100	100	NG_049253.1
5	4368	5024	*qnrS2*	100	100	NG_050544.1

^*a*^ Nucleotide blast with minimum identity thresholds of 80% and 70% and minimum coverage thresholds of 90% and 90%, for PlasmidFinder and ResFinder, respectively.

### The phylogeny of MCRs

**Fig 1:**
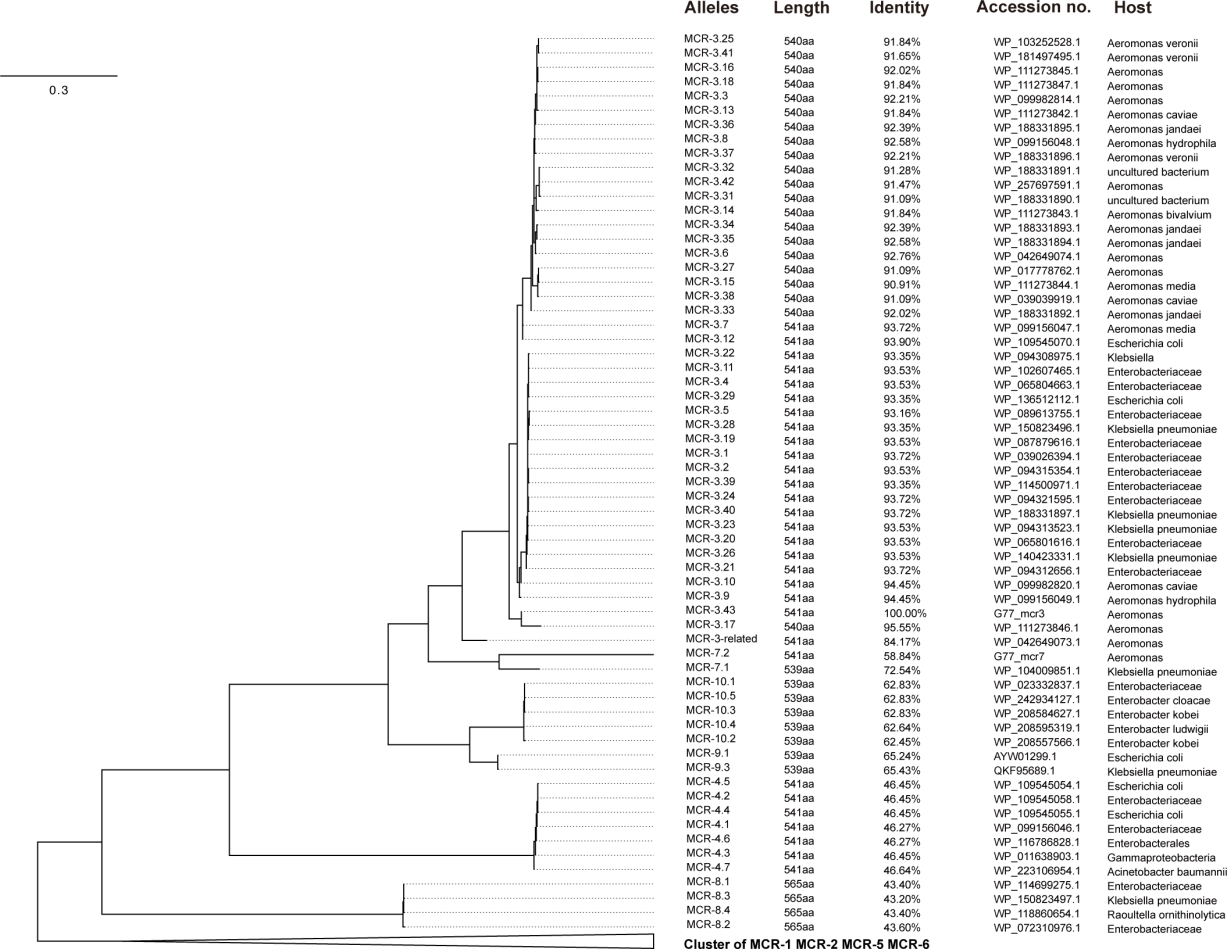
The phylogenetic tree of MCR. The phylogenetic tree was constructed by IQ-TREE 2’s LG + I + G4 model. The cluster of MCR-1, MCR-2, MCR-5, and MCR-6 is collapsed. The column of alleles is the protein ID of MCR. The column of length is the MCR’s amino acid length. The column of identity is the sequences’ identity compared with MCR-3.43 using the blastp. The column of Accession number is the protein’s accession number of MCR. The column of the host is the host genome of MCR.

A few amino acid variations (26 aa) distinguish MCR-3-like from MCR-3.17. However, many amino acid variations (166 aa) distinguish MCR-7-like from MCR-7.1. Phylogenetic analysis of MCRs (MCR-1 to MCR-10) revealed that MCR-3-like formed a distinct cluster with MCR-3.17, sharing a high amino acid identity of ≥90.91% with the reported MCR-3 variants. Consequently, we designated this variant as MCR-3.43. MCR-7-like clustered separately with MCR-7.1, which was distinct from the other MCR variants (Fig. 1). As a result, we designated this variant as MCR-7.2.

### The genetic context of *mcr-3.43*, *mcr-7.2*, *bla*_NDM-1_, and *bla*_KPC-2_

**Fig 2:**
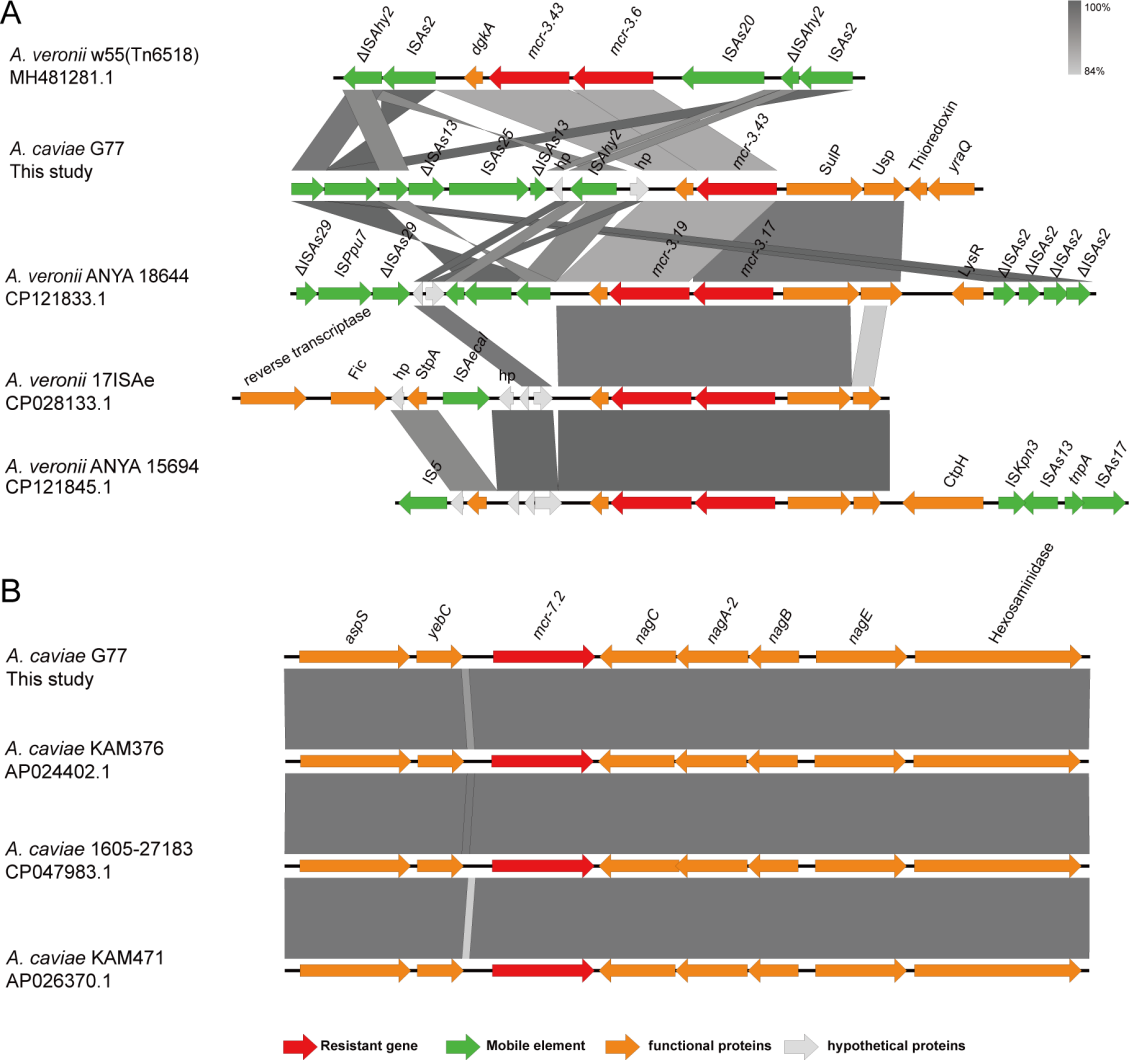
The genetic context of (**A**) *mcr-3.43* and (**B**) *mcr-7.2*. Different-colored arrows indicate different open reading frames (ORFs), while the direction of the arrow indicates its transcription direction. Light gray shadows represent homologous areas.

The two *mcr* variants and *bla*_NDM-1_ were detected on the chromosome of G77, and *bla*_KPC-2_ was found on an IncU-type putative plasmid. Through BLAST analysis, we identified a high similarity between the genetic context of the *mcr-3.43* segment in some *A. veronii* genomes [ANYA 18644 (GenBank accession no. CP121833.1), 17ISAse (GenBank accession no. CP028133.1), and ANYA 15694 (GenBank accession no. CP121845.1)] (*dgkA-mcr-3.19-mcr-3.43*) and G77 (*dgkA-mcr-3.43*). Furthermore, a composite transposon, Tn*6518* (IS*As2*-ΔIS*Ahy2*-IS*As20-mcr-3.6-mcr-3*-like-*dgkA*-IS*As2*-ΔIS*Ahy2*), in *A. veronii* strain w55(MH481281.1), shares a high similarity with the *dgkA-mcr-3.43* segment in G77 (Fig. 2A). Tn*6518* is known to facilitate the transmission of the *mcr-3.6-mcr-3-like* segment (26). The *dgkA-mcr-3.43* segment in G77 was flanked by both hypothetical and functional protein-coding genes. Notably, the *mcr-3.19-mcr-3.43* segment in ANYA 18644, 17ISAe, and ANYA 15694 and the *dgkA-mcr-3.43*-segment in G77 lacked transfer elements in the upstream region but contained two functional genes, SulP (sulfate permease) and Usp (universal stress protein). A complete IS*Ahy2* was located downstream of the *mcr-3.19-mcr-3.17*-like segment in ANYA 18644 and the *dgkA-mcr-3.43* segment in G77, whereas IS*Ahy2* was interrupted into two parts in Tn*6518* detected in *A. veronii* strain w55 (GenBank accession no. MH481281.1). Intriguingly, the downstream regions of IS*Ahy2* in G77 exhibited complete IS*As25* and truncated segments of IS*As13*, distinguishing it from the other strains shown in Fig. 2. Furthermore, the downstream regions of ΔIS*As13* in G77 encoded a complete IS*As2* and a truncated IS*Ahy2*, with the opposite direction compared to that of Tn*6518* and ANYA 18644 (GenBank accession no. CP121833.1).

The *mcr-7.2* gene was found to be chromosomally located in strain G77. We observed that the *mcr-7.2* segment was flanked by functional genes rather than mobile elements, as depicted in Fig. 2B. Through comparative Blast analysis, we discovered a significant similarity between the *mcr-7.2* segment in G77 and three other *A. caviae* strains: KAM376 (GenBank accession number AP024402.1), 1605–27183 (GenBank accession number CP047983.1), and KAM471 (GenBank accession number AP026370.1). Upstream of the *mcr-7*-like gene, we identified the presence of the *aspS* and *yebC* genes. Downstream, a cluster of genes consisting of *nagC-nagA-2-nagB-nagE* was observed, along with the inclusion of a hexosaminidase gene.

**Fig 3:**
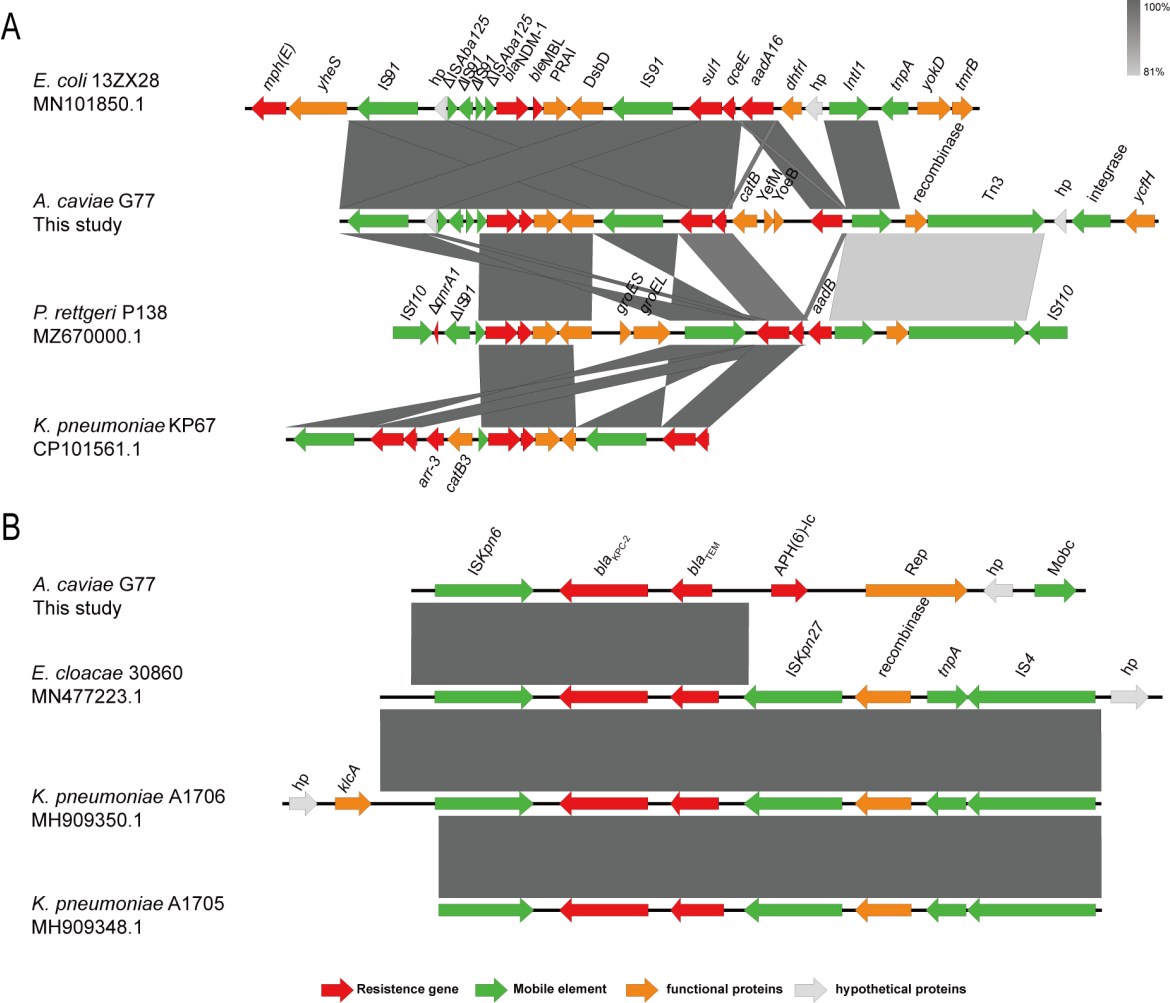
The genetic context of (**A**) *bla*_NDM-1_ and (**B**) *bla*_KPC-2_. Different-colored arrows indicate different open reading frames (ORFs), while the direction of the arrow indicate its transcription direction. Light gray shadows represent homologous areas.

The genetic arrangement of *bla*_NDM-1_ on the G77 chromosome exhibited specific mobile elements located both upstream and downstream. Upstream, we observed the presence of IS*91*, ΔIS*Aba125*, and ΔIS*91* elements, while a complete IS*91* element was identified downstream. The precise genetic context of *bla*_NDM-1_ on the G77 chromosome was represented as "IS*91*-hp-ΔIS*Aba125*-ΔIS*91*-ΔIS*91*-ΔIS*Aba125-bla*_NDM-1_-*ble*_MBL_-PRAI-DsbD-IS*91-sul1-qceE*", which closely resembles the arrangement observed in *E. coli* 13ZX 28 (GenBank accession number MN101850.1). Furthermore, the downstream region of *sul1-qceE* contained an IntI1-recombinase-Tn3 segment, similar to that observed in *Providencia rettgeri* P138 (GenBank accession no. MZ670000.1). However, compared to the genetic context of *bla*_NDM-1_ in *P. rettgeri* P138, the "*groES-groEL*" segment appeared to be truncated in G77 and *K. pneumoniae* KP67 (GenBank accession no. CP101561.1) (Fig. 3A). It is noteworthy that unlike G77, where *bla*_NDM-1_ was integrated into the chromosome, *bla*_NDM-1_ in 13Z × 28, P138, and KP67 was located on plasmids. Based on the genetic context of *bla*_NDM-1_ in G77, we can infer that its origin likely involved plasmid-mediated transmission, facilitated by the integration of IS*91* into the chromosome. Inverse PCR confirmed that no circular intermediate is generated, indicating that the *bla*_NDM-1_ is not acquired by translocatable unit cointegration (27). These findings provide valuable insights into the mechanisms underlying the dissemination of *bla*_NDM-1_ and its potential impact on antimicrobial resistance.

The *bla*_KPC-2_ gene was identified on an IncU plasmid within *A. caviae* G77. Upstream of the *bla*_KPC-2_-*bla*_TEM_ segment in G77, we discovered the presence of a mobile element called IS*Kpn6*, exhibiting sequence similarities with *E. cloacae* 30860 (GenBank accession number MN477223.1), *K. pneumoniae* A1706 (GenBank accession number MH909350.1), and *K. pneumoniae* A1705 (GenBank accession number MH909348.1). However, in contrast to these segments, the region downstream of the *bla*_KPC-2_-*bla*_TEM_ segment in G77 lacked the IS*Kpn27* mobile element. Instead, it was followed by the sequence "APH (6)-Ic-Rep-hp-Mobc." This distinct organization of *bla*_KPC-2_ and *bla*_TEM_, coupled with the presence or absence of specific mobile elements, underscores the potential genetic diversity associated with carbapenem resistance genes among different bacterial strains (Fig. 3B). A comprehensive understanding of these genetic variations is critical to unraveling the dynamics of antibiotic resistance dissemination and formulating effective countermeasures.

### Expression of *mcr-* and carbapenemase-encoding *genes* under colistin and meropenem stress in *A. caviae* G77

**Fig 4:**
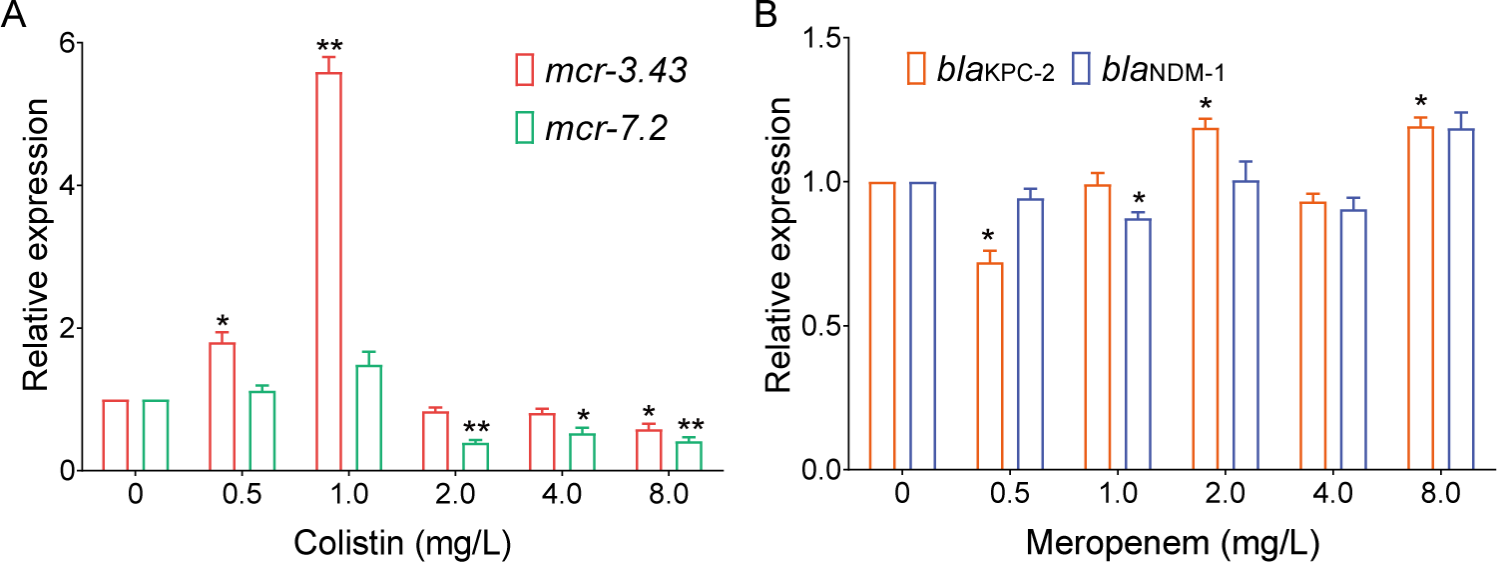
Gene expression with and without antibiotic exposure (colistin and meropenem). Fold changes in mRNA levels were determined by RT-qPCR. The data have been normalized to values for reference *rpoD* gene. The experimental groups included LB supplemented with 0.5, 1, 2, 4, and 8 mg/L of colistin or meropenem, and antibiotic-free LB was used as control. The data are shown as the means ± SD of the results from three individual assays. Statistical signiﬁcance was determined by Student’s *t*-test relative to the CI for competition between experimental groups versus control; *P < 0.05; **P < 0.01; ***P < 0.001.

**Fig 5:**
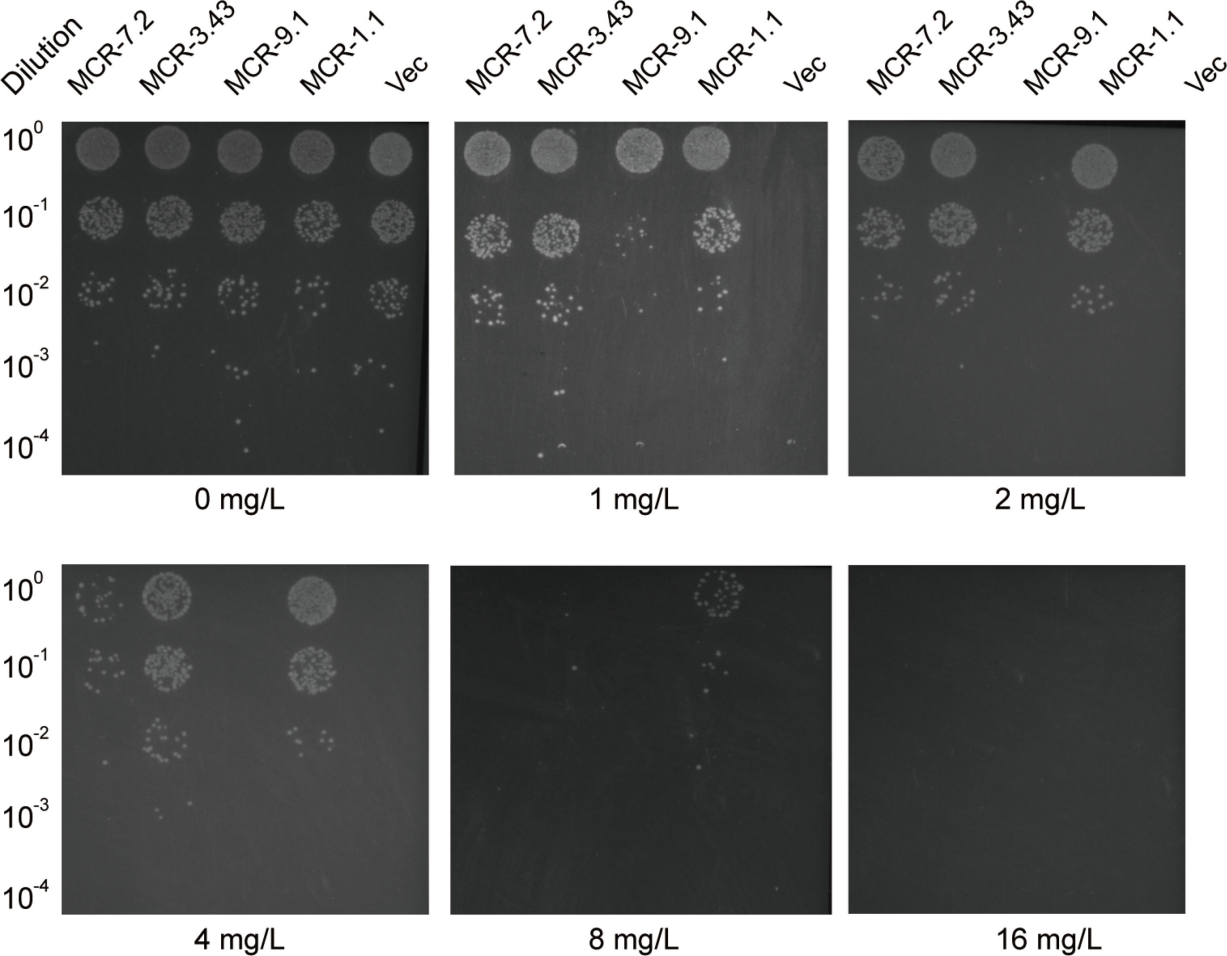
Growth viability of *E. coli* harboring different versions of MCR family of enzymes. The isolates were grown on the LBA plates supplied with varied levels of colistin. Designation: Vec, pBAD33.

Induction assays were performed using different concentrations of colistin and meropenem to evaluate the expression of *mcr-3.43*, *mcr-7.2*, *bla*_KPC-2_, and *bla*_NDM-1_ in *A. caviae* G77. Compared to the untreated control group, *mcr-3.43* showed upregulated expression in the colistin 0.5 (*P* < 0.05) and 1 mg/L (*P* < 0.01) groups and downregulated expression in the colistin 8 mg/L group (*P* < 0.05). In contrast, compared with the untreated control group, the transcript level of the *mcr-7.2* gene was induced when bacteria were inoculated with 0.5 and 1 mg/L of colistin while repressed when colistin was ≥2 mg/L (*P* < 0.05). In contrast, the expression of *bla*_KPC-2_ and *bla*_NDM-1_ shows no significant difference with less than twofold change when treated with a series of meropenem (Fig. 4). The divergence of MCR enzymes exists in the context of antibiotic resistance, as demonstrated by the colistin killing assay (Fig. 5). To test the activity of *mcr-3.43* and *mcr-7.2*, the coding regions of *mcr-3.43*, *mcr-7.2*, *mcr-9.1*, and *mcr-1.1* (positive control) were cloned into pBAD vector and expressed in *E. coli* Top10. The strains were incubated with twofold colistin dilutions (from 0 to 16 mg/L). *mcr-3.43* expression conferred colistin resistance up to 8 mg/L, but the resistance was weaker than that of *mcr-1*. Meanwhile, *mcr-7*.2 expression conferred colistin resistance at 4 mg/L, and *mcr-9.1* conferred resistance only at 1 mg/L. The data suggest that the activity of *mcr-3.73* against colistin is higher than that of *mcr-7.2*, and it may play a more critical role in colistin resistance.

### Plasmid transformability and stability

Conjugation assays showed that pKPC2 could not be successfully transferred into EC600 successfully by conjugation, albeit multiple attempts were made.

To determine the stability of the *bla*_KPC-2_ plasmid, a plasmid stability experiment was performed. The retention rate of *bla*_KPC-2_ in the G77 isolate was 100% after 10-day passages, suggesting that pKPC2 is stable in G77. In addition, the plasmid is a low-copy-number plasmid, with 1.02 ± 0.08 copy/CFU at day 0 and 1.06 ± 0.07 copy/CFU at day 10, with no significance.

## DISCUSSION

The genus *Aeromonas* encompasses a wide range of aquatic environments and is associated with both intestinal and extra-intestinal infections in humans and animals (6). However, the emergence of multidrug-resistant organisms, particularly carbapenem-resistant organisms (CRO) (28), represents a formidable global challenge. Colistin, a last-resort antimicrobial agent, remains one of the limited treatment options for severe CRO infections. Unfortunately, the alarming increase in CRO strains with acquired colistin resistance has now become a worldwide phenomenon, posing a significant threat to the efficacy of colistin therapy (29). The IS-mediated *mcr* colistin resistance genes are of particular concern, which are capable of horizontal transfer between different species and spreading across continents. This represents a specific and significant threat to public health and clinical management on a global scale (28). In the ongoing battle against antimicrobial resistance, continuous surveillance and monitoring play a pivotal role in upstanding the mechanisms of resistance transmission and formulating effective strategies to combat this escalating problem (30). In this way, we can protect public health and ensure the continued effectiveness of antimicrobial treatments for present and future generations.

This study identified a colistin-resistant strain of *A. caviae*, designated as G77, which carries multiple antibiotic resistance genes (ARGs). Through comprehensive whole-genome sequencing, we revealed the presence of novel genes, including *mcr-3.43*, *mcr-7.2*, *bla*_NDM-1_, and *bla*_KPC-2_, within the G77 strain. Notably, *bla*_KPC-2_ was specifically located on an IncU plasmid. Remarkably, this is the first documented case of all four genes coexisting within a single *Aeromonas* strain. Of particular interest, MCR-3.43 shares the highest amino acid identity (95.55%) with MCR-3.17, while MCR-7.2 shares the highest amino acid identity (68.68%) with MCR-7.1. These findings provide valuable insights into the emergence of multidrug-resistant strains within the *Aeromonas* genus and highlight the potential for widespread dissemination of antibiotic resistance within this group. These important discoveries contribute to the understanding of the genetic dynamics underlying multidrug resistance in *Aeromonas* species. Such knowledge is critical to addressing the global challenge of antibiotic resistance and developing effective strategies to mitigate its impact.

A comparative analysis investigated the relationship between the newly identified MCRs and the known MCR variants (MCR-1 to MCR-10). The analysis reveals that MCR-3.43 and MCR-3.17 show only a few amino acid variations (26 aa), while MCR-7.2 and MCR-7.1 exhibit a substantial number of amino acid variations (166 aa). MCR-3.43 forms a distinct cluster alongside MCR-3.17 and shares a high amino acid identity of ≥90.91% with the known MCR-3 variants. Consequently, the newly discovered protein is designated as MCR-3.43. Furthermore, phylogenetic analysis demonstrated that MCR-7.2 also forms a separate cluster with MCR-7.1, exhibiting a moderate amino acid identity of 68.89%. Notably, MCR-7.2 and MCR-7.1 stand out from other MCRs in the phylogenetic tree. These findings provide valuable insights into the genetic relationships and variations among different MCR variants, enhancing our understanding of their diversity and evolution.

We discovered that *mcr-3.43* is located adjacent to IS*Ahy2* and shares several elements similar to Tn*6518*, suggesting its potential mobility. In contrast, *mcr-7.2* is surrounded by a few functional genes and is situated on the chromosome. Interestingly, the identical genetic context of *mcr-7.2* found in *A. caviae* suggests a limited dissemination of this gene thus far. Moving on to the G77 strain, the *bla*_NDM-1_ segment is on the chromosome, flanked by two IS*91* elements. Conversely, *bla*_KPC-2_ is positioned on an IncU plasmid adjacent to a complete IS*Kpn6*. These observations suggest that IS elements facilitate the transmission of the two carbapenemase genes. Understanding the genetic arrangements and mobile elements associated with resistance genes is crucial for comprehending their dissemination and devising effective strategies to combat antibiotic resistance. These findings contribute significantly to our knowledge of the genetic characteristics and potential transmission mechanisms of resistance genes in *A. caviae*.

In conclusion, our study highlights that *A. caviae* is an essential reservoir of colistin-resistant genes and carbapenemase genes in hospital wastewater, which could be a risk for the wide dissemination of these genes in the future. Developing robust surveillance strategies and effective countermeasures is urgent for the prevention of such reservoir dissemination.

### Limitation of the study

This study is limited by its reliance on a single strain of *A. caviae* isolated from hospital sewage. Findings need to be completed in terms of both diversity and prevalence of resistance genes in *Aeromonas* spp. We recommend further research, including broader sampling and functional analysis, to promote a more comprehensive grasp of colistin and carbapenem resistance in *Aeromonas* spp. and other relevant pathogens.

## MATERIALS AND METHODS

### Sample collection, *mcr*-positive bacterial identiﬁcation, and susceptibility testing

MCR-producing bacteria were recovered from wastewater samples collected in a tertiary hospital in September 2020. Water samples were spread on Luria–Bertani (LB) (Sangon Biotech, Shanghai, China) agar plate supplemented with colistin (4 mg/L) for obtaining colistin-resistant bacteria. The species identification was performed using MALDI-TOF Mass Spectrometry (Hexin Instrument Co., Ltd, Guangzhou, China). The presence of *mcr*-like genes (*mcr-1* to *−10*) was investigated by PCR analysis using speciﬁc primers (31). Antimicrobial susceptibility was evaluated following CLSI guidelines, and the results were interpreted according to CLSI instructions (M100-S30, 2020) (https://clsi.org/standards/products/microbiology/documents/m100/), except that polymyxin B, colistin, and tigecycline resistance was deﬁned according to EUCAST (version 10.0) (https://eucast.org/clinical_breakpoints/) clinical breakpoints. *Escherichia coli* ATCC 25922 was used as the quality control strain.

### Whole-genome sequencing (WGS), assembly, and bioinformatic analysis

Genomic DNA was extracted from G77 using a Gentra Puregene Yeat/Bact.Kit (Qiagen, San Francisco/Bay area, CA, USA) and subjected to WGS on Illumina novaseq 6000 platform (Illumina, San Diego, CA, USA) and Nanopore PromethION platform (Nanopore, Oxford, UK). The hybrid assembling was performed using Unicycler v0.4.8 (32), and the assembly was annotated by Prokka v1.17 (33). The contigs were completely circularized using PCR and Sanger sequencing.

Identiﬁcation of antibiotic resistance genes were carried out via the Center for Genomic Epidemiology website (http://www.genomicepidemiology.org/). Plasmid replicon types were identified using the Mob-suite (https://github.com/phac-nml/mob-suite). The species of the isolates was confirmed via KmerFinder(https://cge.food.dtu.dk/services/KmerFinder/). The insertion sequences (ISs) were annotated using ISfinder (https://isfinder.biotoul.fr/). Resistance genes were annotated using resfinder (https://cge.food.dtu.dk/services/ResFinder/). The genetic environments were visualized by Easyﬁg 2.2.2 (34). The sequence of *bla*_KPC-2_-harboring plasmid was blasted against the previously reported plasmids and NCBI, and plotted by BLAST Ring Image Generator (BRIG). The presence of strains harboring *mcr* genes was screened by blasting the sequences in NCBI identical protein groups (IPGs) database. All the published *mcr* genes in the IPGs database were downloaded to build a phylogenetic tree with *mcr-3.43* and *mcr-7.2* in this study. The phylogenetic tree was constructed by IQ-TREE 2’s LG + I + G4 model (35).

The complete genome sequence of G77 has been deposited in GenBank under accession no. JAVHYK000000000.

### Functional evaluation of *mcr-3.43* and *mcr-7.2*

The coding regions of *mcr-3.43* and *mcr-7.2* were amplified from G77 and ligated with pBAD vector and expressed in *E. coli* Top10. *E. coli* Top10 harboring *mcr-1.1* and empty pBAD vector previously constructed by our team (31) were employed as the positive and negative controls, respectively. The colistin killing assay was performed as previously described (36). In brief, different strains were incubated at 37°C to an optical density (OD_600_) value of 0.3 to 0.4 and were then diluted 1:100 in LB broth, respectively. Fifty microliters of the diluted cultures was mixed with 50 µL of colistin dissolved in a phosphate-buffered saline (PBS) solution and was placed in a 96-well plate to ensure the final colistin concentrations of 0, 1, 2, 4, 8, and 16 mg/L. After 1 h of incubation at 37°C with aeration, the cultures were serially diluted in PBS, and 5 µL each was plated onto LB agar plates.

### Antibiotic induction, bacterial RNA Isolation, and quantitative real-time PCR (RT-qPCR)

For induction assays, G77 was grown in LB without antibiotic until OD_600_ = 0.5. Overnight culture of the bacteria was diluted in fresh LB broth (1:1,000, vol/vol) with final colistin or meropenem concentrations of 0.5, 1, 2, 4, and 8 mg/L. The antibiotic-free medium was used as control. The culture was incubated at 37°C for 30 min and harvested by centrifugation at 4°C. RNA was extracted using RNeasy Mini Kit (Qiagen, Valencia, CA, USA) according to the manufacturer’s instructions, followed by genomic DNA elimination. cDNA was synthesized using the PrimeScript RT Reagent Kit with gDNA Eraser (Takara Bio USA). gDNA removal was confirmed using PCR. RNA size, integrity, and total amount were determined using a NanoDrop 2000 spectrophotometer (Thermo Fisher Scientific, MA, USA).

The primers used for RT-qPCR were designed using Primer Premier 6.0 and are listed in Table S1. The ampliﬁcation efﬁciency of all primer pairs was tested using standard dilution procedures. RT-qPCR analysis was conducted on an Applied Biosystems ViiA 7 sequence detection system with SYBR green ﬂuorescence dye. The 16S rRNA gene was used as a reference control for normalization. The relative differences in gene expression were calculated as a fold change using the formula 2^−ΔΔCT^ (Livak and Schmittgen, 2001). Expression changes > twofold with *P* < 0.05 were considered statistically signiﬁcant.

### Stability and transferability of the *bla*_KPC-2_-carrying plasmid

The plasmid stability was conﬁrmed as previously described with minor modifications (37). Brieﬂy, single colonies were enriched in 5 mL of LB broth at 37°C for 24 h. The suspension was then inoculated with 1:1,000 dilution in antibiotic-free LB broth. The freshly inoculated culture constituted time point one. Isolates were cultured at 37°C in a shaking bath (200 rpm) and serially passaged for 10 days. Cultures were diluted and plated onto antibiotic-free LB plates every 24 h. A total of 50 resulting colonies were randomly selected for PCR assays to determine the proportion of *mcr* or carbapenemase-positive bacteria in each population. Plasmids were considered stable when the retention rates were still over 80% at the end of the experiment. Plasmid copy number was estimated by qPCR according to the previous study (31).

Conjugation experiments were performed using the rifampicin-resistant *E. coli* EC600 recipient strain. Both donor and recipient strains were cultured in exponential phase and then mixed at a 1:1 ratio. The transconjugants were selected on agar plates supplemented with 4 mg/L of meropenem plus 100 mg/L of rifampicin. PCR was performed to conﬁrm the presence of *bla*_KPC-2_ in transconjugants. Positive transconjugants were validated using Vitek MS to confirm the species.

## SUPPLEMENTAL MATERIAL

Table S1 spectrum.03685-23-s0001.docx Primers used in this study.

Table S2 spectrum.03685-23-s0002.xlsx Information of bacteria isolated from seweage water.

ASM does not own the copyrights to Supplemental Material that may be linked to, or accessed through, an article. The authors have granted ASM a non-exclusive, world-wide license to publish the Supplemental Material files. Please contact the corresponding author directly for reuse.
