# Supplementary material for: First report of multidrug-resistant carbapenemase-producing Aeromonas caviae co-harboring mcr-3.43 and mcr-7.2
Source: Microbiol Spectr. 2024 Mar 21;12(5):e03685-23. doi: 10.1128/spectrum.03685-23 (PMC11064524; doi:10.1128/spectrum.03685-23)
Supplement: Table S1 — Primers used in this study. [file spectrum.03685-23-s0001.docx]

**Table S1.** Primers used in this study.

| **Target** | **Primer name** | **Sequence (5’-3’)** | **Product size (bp)** | **Source** | |
| --- | --- | --- | --- | --- | --- |
| **Gene identification** | | | | | |
| *mcr-3.17* | *mcr-3.17*-F | ATGCCTTACATTTTGAAAATTAA | 1627 | This study | |
|  | *mcr-3.17*-R | TTATTGAACATTACGACATTG |  |  |  |
| *mcr-7* | *mcr-7*-F | ATGCGTCTCACTCTGGGAG | 1627 | This study | |
|  | *mcr-7*-R | TCACCCCGCCCGGCACGG |  |  |  |
| *bla*_KPC-2_ | KPC-F | ATAACGCCGCCGCCAATT | 431 | This study | |
|  | KPC-R | GCTGTGCTTGTCATCCTTGTT |  |  |  |
| *bla*_NDM-1_ | NDM-F | AGCTCGCACCGAATGTCTG | 342 | This study | |
|  | NDM-R | CATTGGCGGCGAAAGTCAG |  |  |  |
| **Complete circular confirmation** | | | | |  |
|  | *G77-**plas1*-F | TCATGGAGCAAAGTAGTCG | 608 | This study | |
|  | *G77-plas1*-R | ATCAGTTTGTCCACCTTTG |  |  |  |
|  | *G77-plas2*-F | TAGATGGCGAAACTGTAGTG | 710 | This study | |
|  | *G77-plas2*-R | GTCGTTTTTGGGCGTGTAT |  |  |  |
|  | *G77-plas3*-F | GCAAAAGAGAACTCCAGGG | 731 | This study | |
|  | *G77-plas3*-R | GGCCAGCAGATAGCCAAG |  |  |  |
|  | *G77-plas4*-F | CGCGGCCAAGACTGGCAG | 694 | This study | |
|  | *G77-plas4*-R | GGCCGTCTCGATGGACAG |  |  |  |
|  | *G77-plas5*-F | ACTCAGGCCTTAACCTGG | 708 | This study | |
|  | *G77-plas5*-R | CAGGATGGGAGATAACGC |  |  |  |
| **Gene expression** | | | | |  |
| *mcr-3.17* | G77-PBAD-MCR3.17-F | ACTTCCAGGGCTCGAGAGATCCGATGCCTTACATTTTGAAAATTAA | 1667 | This study | |
|  | G77-PBAD-MCR3.17-R | GCCAAGCTTCGAATTCATTATTGAACATTACGACATTG |  |  |  |
| *mcr-7* | G77-PBAD-MCR7-F | ACTTCCAGGGCTCGAGAGATCCGATGCGTCTCACTCTGGGAG | 1667 | This study | |
|  | G77-PBAD-MCR7.1-R | GCCAAGCTTCGAATTCATCACCCCGCCCGGCACGG |  |  |  |
| **Translocatable unit confirmation** | | | | | |
| *bla*_NDM-1_ | TU-F | CATGCCTAGCATTCACCTTC | ~3000 | This study | |
|  | TU-R | GATCAATAGGCGTCGTTCAG |  |  |  |
| **RT-qPCR** | | | | |  |
| *mcr-3.43* | Mcr3.43-F | AGCTGGTTGGCTTCCACCTGAT | 162 | This study | |
|  | Mcr3.43-R | CCGTGTAGCGGATGGTGTTGTC |  |  |  |
| *mcr-7-like* | G77-MCR7-like-F | CTGGTCCTGCTGTGGGTGAAGA | 241 | This study | |
|  | G77-MCR7-like-R | AGGGAATGGGAGTGGCGAAGAC |  |  |  |
| *rpod* | G77-RPOD-F | TTCCGTCAATTCCGCCTGATGC | 170 | This study | |
|  | G77-RPOD-R | TCGCACTCGTTGTTGGTGAAGG |  |  |  |
| **Gene copy number** | | |  |  | |
| *incU* | *incU*-F | GAGGCATCTTTTCTGGCGTC | 164 | This study | |
|  | *incU*-R | CCTCATAGGATTTCGCCTTGC |  |  |  |
